# Literary Gossip and Record

**Published:** 1862-07

**Authors:** 


### Art. XI.—LITERARY GOSSIP AND RECORD.

### THE ANTIQUITY OF PLAGUE.

M. Chabas* has recently thrown additional light upon the probable antiquity of plague. In a commentary on the hieroglyphical denominations of the *Hycsos,* or Shepherd-Kings of Egypt, he suggests a new reading, of great interest to the medical historian, for one of the several contumelious names which the Egyptians made use of when referring to them. The name in question is AAT-U. M. Chabas had previously translated the term *barbarians;* Mr. Goodwin, *invaders, revolters;* and M. Brugsch, *rebels, insurgents.* Each gentleman, however, wished it to be understood that these interpretations did not convey the intimate sense of the word. Now, in certain papyri in which the phrase AAT-U is met with, it evidently signifies something deadly. Thus, in one celebrating the triumphs of Rameses II., we read—"He made Khita a habitation for the dead, as though he were Pakht exerting his fury in the track of the AAT-U." But in the Leyden papyri more explicit texts are found.

---

* *Melanges Egyptologiques.* Par F. Chabas. A Chalon-sur-Saone. 1862.

These manuscripts, which were discovered at Memphis, rolled the one above the other, are books of magical formulæ. The preservative effects of some of these are announced in these terms :—" *The man is preserved from the annual Aat, the enemy will not seize upon him.*" " *The annual Aat will not prostrate him :*" " *Weakness will not master him, neither the annual Aat kill him, nor sickness destroy him.*" From these formulæ, in which AAT is accompanied by the word TER (*yearly*), M. Chabas is of opinion, that the term refers to some periodical and very formidable plague, since the Egyptians sought to defend themselves from its attack by magical means, as they were accustomed to protect themselves from crocodiles, ferocious animals, and venomous reptiles. Further, there is a passage in the calendar of Sallier which reads thus :—" *The air in the heaven this day is mingled with the annual Aats ;*" or, " *The annual Aats now mingle with the atmosphere.*"

The AAT, then, M. Chabas infers, was a contagious or epidemic malady, the germs of which were transported by the air we breathe. Now, it is noteworthy that the passage cited from the calendar refers to the 19 Tobi, that is to say, to the first month following the retreat of the waters of the inundation. Hence, M. Chabas identifies the AAT with the plague, which he asserts manifests itself in Egypt precisely at this epoch.

He is well aware that it is common to attribute to the climate of ancient Egypt a constant salubrity, and to assert as the principal and very modern cause of plague, the cessation of the custom of embalming the dead at the time of the introduction of Christianity. But against the evidence of Herodotus, who speaks of the healthy climate and regularity of the seasons of Egypt, it is only necessary to oppose the fact of the pestilence which ravaged that country a few years after the voyage of the historian, and which afterwards spread itself over Asia and Europe. It is not easy to admit that this frightful calamity was the first manifestation of a plague new to Egypt. Even before the time the country was civilized, the Nile covered it each year as now with its fertilizing mud, from which has always emitted insalubrious emanations under the influence of the sun. That under the administration of the Pharaohs, thanks to a perfect system of irrigation, the plague was less frequent, is possible, but it was not the less known and feared by the Egyptians.

"The Scripture often mentions pest, under the name of דבר," continues M. Chabas ; " with the sword and the famine, it proved the terrible triad of mortal plagues with which the unfaithful Israelites and the enemies of the people of God were menaced. The pestilence which punished the crime of David carried off 70,000 Israelites in three days. In speaking to Pharaoh of the pestilence with which God would strike the Hebrews if they did not sacrifice in the desert, Moses certainly mentioned a plague known to the Egyptians, and well fitted to make a lively impression upon their minds.

"That the Egyptian name of the pest, as its Hebrew name, was applied to divers destructive plagues, cannot be doubted. In the hieroglyphics particularly, AAT-U designates the executors of celestial chastisements, *who march in the track of the gods, striking off heads and breaking necks.* And this tropical extension of the meaning of the word pest in no degree enfeebles the terrible energy of the expression. Now, we have demonstrated that the Egyptians, in order to characterize their enemies, applied to them epithets which represented them as extended without life and force at their feet. Is it not remarkable that with regard to the Shepherd-Kings they have made use, on the contrary, of a word which, far from recalling an idea of contempt and triumph, awakes a sentiment of extreme terror and hate?

"If my philological deductions are admitted, we have acquired a significant commentary on the report of Manetho concerning the cruelty of the Shepherd-Kings, and the disasters with which they had struck Egypt. In the same time we have obtained an historical result in the demonstration of the great antiquity of the pest, contrary to the opinions most generally admitted even at this day."

